# Effect of Aging on Intraventricular Kinetic Energy and Energy Dissipation

**DOI:** 10.3390/jcdd10070308

**Published:** 2023-07-19

**Authors:** Donato Mele, Riccardo Beccari, Gianni Pedrizzetti

**Affiliations:** 1 Department of Cardiac Thoracic Vascular Sciences and Public Health, University of Padova, 35128 Padova, Italy; riccardo.beccari@studenti.unipd.it; 2 Department of Engineering and Architecture, University of Trieste, 34127 Trieste, Italy; giannip@dia.units.it

**Keywords:** aging, blood speckle imaging, echocardiography, energy loss, 4D flow cardiac magnetic resonance, HyperDoppler, kinetic energy, kinetic energy dissipation, cardiac ultrasound, vector flow mapping

## Abstract

In recent years, analysis of kinetic energy (KE) and the rate of kinetic energy dissipation (KED) or energy loss (EL) within the cardiac chambers, obtained by cardiac imaging techniques, has gained increasing attention. Thus, there is a need to clarify the effect of physiological variables, specifically aging, on these energetic measures. To elucidate this aspect, we reviewed the literature on this topic. Overall, cardiac magnetic resonance and echocardiographic studies published so far indicate that aging affects the energetics of left and right intraventricular blood flow, although not all energy measures during the cardiac cycle seem to be affected by age in the same way. Current studies, however, have limitations. Additional large, multicenter investigations are needed to test the effect of physiological variables on intraventricular KE and KED/EL measures.

## 1. Introduction

In recent years, analysis of kinetic energy (KE) and the rate of kinetic energy dissipation (KED) or energy loss (EL) within the cardiac chambers has become possible using different cardiac imaging techniques, such as four-dimensional (4D) flow cardiac magnetic resonance (CMR), contrast echocardiography with particle imaging velocimetry (Echo-PIV), and color Doppler-based ultrasound techniques [1]. These measures are of potential interest in cardiology since they potentially represent a new way to observe cardiac function and workload [1]. Analysis of KE and KED/EL has been applied to characterize athletes’ heart [2,3,4] and to patients with renal disease [5,6], prediabetes and type 2 diabetes mellitus [7], systemic lupus erythematosus [8], aortic regurgitation [9], aortic stenosis [10], cardiac amyloidosis [11], hypertrophic cardiomyopathy [12], myocardial infarction [13,14], and heart failure [15,16,17]. Although these applications are only preliminary, they clearly show the attention of clinical investigators toward the information coming from intracardiac flow dynamics analysis.

Unfortunately, different cardiac imaging techniques are not interchangeable in evaluating KE and KED/EL since such measures depend on image properties—primarily on spatial resolution—and each one of these techniques has its own advantages and limitations in this sense [1]. Thus, it should not be a surprise if the values of the same measures provided by these techniques do not overlap in the same subjects or patients [18]. Conversely, the effect on KE and KED/EL by physiological variables, such as age, sex, ethnicity, heart rate, blood pressure, and exercise, is expected to be independent of the technique used to obtain these measures.

Age, sex, and ethnicity are all unmodifiable variables. All of them determine differences in cardiac size and wall thickness [19]. Age affects cardiac function essentially through modifications of cardiac and myocardial structure, especially at younger and older ages [20,21]. The effect of aging also occurs in patients of different genders and ethnicities. In brief, age significantly influences many cardiac evaluations and heart performance [21,22]. Early recognition of cardiac alterations due to aging could be of clinical interest because there are emerging treatments that target age-related pathways [23,24,25,26,27].

In this review article, we sought to explore whether aging influences KE and KED/EL in the same way by using CMR, Echo-PIV, and non-contrast ultrasound techniques in normal subjects. This information should provide the basis not only to understand how to properly use these techniques and measures but also to address future lines of experimental and clinical research in the field.

## 2. Kinetic Energy and Energy Dissipation: Basic Principles

The fluid energy of blood flow moving in the cardiac chambers consists of potential energy, imputable mainly to pressure, and KE. Intracardiac KE is a nondirectional quantity and depends on blood flow velocity and density. KE and potential energy can convert to each other and transform into inertia during the accelerative/decelerative phases of the cardiac cycle. In addition, part of the KE (i.e., the KED/EL) is dissipated by viscous friction and turbulence during the various phases of the cardiac cycle, which is an irreversible loss of the total fluid energy. In general, the higher the energy being dissipated, the lower the efficiency of the system.

Similar to KE, KED/EL also depends on blood flow velocity, which has been documented previously by several authors [28,29]. In healthy individuals, the EL at the time of the left ventricular (LV) early diastolic filling has been shown to correlate directly to the peak E-wave blood velocity through the mitral valve [28]. Mean diastolic EL also correlates with peak E-wave velocity [29]. In other words, the greater the peak E-wave velocity, the greater the available KE, and the greater the KED/EL during diastole.

## 3. Kinetic Energy and Energy Dissipation: Techniques for Measuring

### 3.1. Cardiac Magnetic Resonance

The 4D flow CMR enables the acquisition of blood flow within a volume of interest in three spatial directions simultaneously. It overcomes the limitation of conventional 2D phase-contrast CMR, in which flow is studied manually by aligning a single velocity-encoding direction with target flow signals [30]. Using 4D flow CMR, KE is calculated from the velocity data. Specifically, based on Newton’s second law of motion, blood flow KE can be calculated using the equation:

KE = 1/2 × Mass × Velocity^2^ (1)

where mass = mean density of blood (1.06 g/cm^3^) × voxel volume.

There are different 4D flow CMR methods proposed by different groups for deriving KE. Eriksson et al. chose a single time point (isovolumetric contraction) and then tracked particles forward and backward through the cardiac cycle to calculate KE [31]. In contrast, Carlsson et al. [32] and Wong et al. [33] calculated instantaneous KE from the velocity data based on cine or manual segmentations of the ventricle. It has been stated that all techniques produce KE values that are very similar and appear equally valid [33].

KE can be measured by 4D flow CMR as an absolute (in μJ) or indexed (KEi) value. Generally, when evaluating the LV or the right ventricle (RV), indexation is performed in relation to the size of the ventricle, expressed by the end-diastolic volume (KEi_EDV_, in μJ/mL). Normalization of KE to EDV reduces the preload dependence of KE because the increased ventricular dimensions will cause the KEi_EDV_ to decrease [13]. Other studies have normalized the LV KE to body surface area (BSA) [34], stroke volume [35], and cardiac output [36]. This, however, precludes direct comparison of non-indexed KE values.

As previously mentioned, blood flow KE is partly dissipated to heat by viscous friction and turbulence. Viscous EL represents the KE being dissipated as a result of frictional forces between blood flow elements, due to the variability of flow velocities within a vessel, or between blood elements and the ventricular wall. It is quantified by 4D flow CMR by providing the three-directional flow velocity field and using the Navier–Stokes equations [37,38]. Turbulent KE (TKE) refers to the KE being dissipated into small turbulent eddies. It is estimated by 4D flow CMR on the basis of intravoxel velocity distribution and its relationship with the MR signal [37,38].

### 3.2. Cardiac Ultrasound Techniques

Using cardiac ultrasound, KE and KED/EL can also be measured. This can be conducted by utilizing the contrast-based-HyperFlow technique (Echo-PIV) and non-contrast ultrasound techniques, such as Vector Flow Mapping (VFM), HyperDoppler, and Blood Speckle Imaging (BSI). In general, using ultrasound techniques, KE cannot be measured with the same accuracy as CMR due to the limited information about the spatial distribution of flow velocity in the LV. Conversely, ultrasound KED/EL seems to be a more reliable measure compared to KE and is, therefore, generally utilized.

VFM uses both color Doppler and speckle tracking images to obtain EL [39,40]. The velocity vectors of each pixel are calculated using the continuity equation starting from both the left- and right-side boundaries and combining the results according to a weight function [39,40]. VFM measures EL as an absolute value in Watts (W) to denote energy transfer over time. However, since the VFM analysis is based on 2D flow assumptions, the EL derived from VFM is expressed per 2D-echo flow distance in W/m.

**Figure 1:**
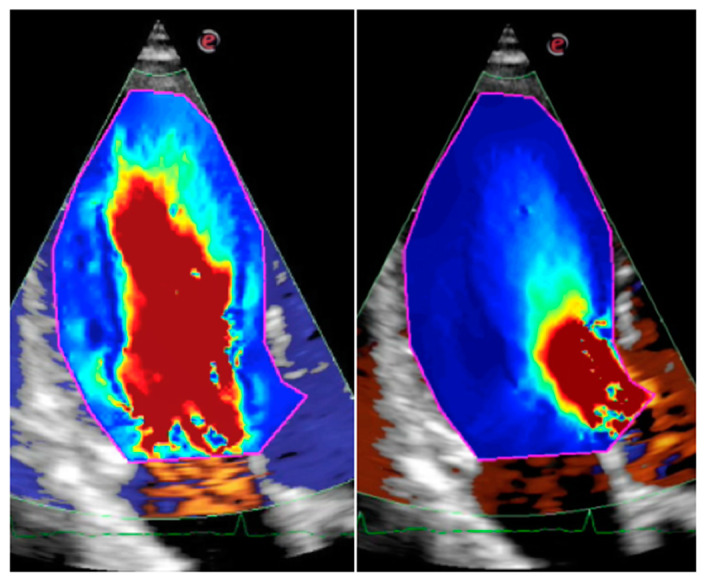
Apical long-axis view of a young normal subject showing the kinetic energy within the left ventricle using the HyperDoppler technique. Blood with the highest kinetic energy is displayed in red color. **Left**: Early diastole (rapid filling). **Right**: peak of systolic ejection.

The HyperDoppler technique is also based on color Doppler information but with different assumptions with respect to VFM [2]. Both the HyperDoppler and the EchoPIV HyperFlow techniques [41] may evaluate LV KE (Figure 1) and KED. This latter is the integral over the LV cavity and over the heartbeat of the rate of KE dissipation (double scalar product of deformation and stress tensors). LV KED is expressed as an indexed measure, which is obtained by dividing KED by the average KE. Therefore, KED is a dimensionless ratio. Normalization, using the average KE, avoids direct dependence on LV KED from the LV size.

Using the HyperFlow and HyperDoppler techniques, other energetic measures can be obtained–for example, the KE fluctuation, which is defined as the standard deviation of the KE, normalized by the corresponding average value [42]. KE fluctuation is informative of the degree of regularity in the flow (or, loosely speaking, of turbulence).

BSI is based on the tracking of the speckles generated by the moving blood cells [43,44]. This allows direct assessment of 2D blood velocity vectors without the mathematical assumptions needed by color Doppler flow mapping. However, because of the high rate of decorrelation of the moving blood speckles, the acquisition frame rate must be very high; thus, BSI is currently only possible with pediatric and transesophageal probes.

Inherent technical limitations of ultrasound techniques may result in inaccuracies in measuring certain parameters. For example, the EchoPIV technique underestimates velocity values and is, therefore, inappropriate for measuring KE, which is dominated by high velocities [45].

## 4. Kinetic Energy and Energy Dissipation: Specific Measures

The KE and KED/EL evaluated by CMR and cardiac ultrasound are generally spatially-integrated measures (over the entire LV size). They can be expressed as instantaneous values at specific times or averaged during relevant phases of the cardiac cycle.

During diastole, the most used evaluations are those conducted at the time of the early LV filling (peak E-wave KE and KED/EL) and atrial contraction (peak A-wave KE and KED/EL) as well as during the entire diastolic phase (mean diastolic KE and KED/EL). Energy measures can also be performed during systole (peak systolic and mean systolic KE and KED/EL) and throughout the cardiac cycle (systo–diastolic or global KE and KED/EL). Having in mind that the separation between systole and diastole is not sharp when seen in terms of fluid dynamics, the HyperFlow and HyperDoppler software analyze flow velocities during an entire heartbeat by using a steady-streaming approach [2,41]; thus, the KED estimated in this way is the global one (gKED).

Compared to 4D flow CMR, echocardiographic techniques evaluate 2D KE and KED/EL. However, flow is three-dimensional, especially in the presence of turbulence, meaning the KED/EL may not be fully measured by 2D echocardiography. For this reason, ultrasound-based techniques utilize, when studying the LV, an apical long-axis view, which contains most of the information on the intracardiac flow dynamics, due to the simultaneous presence of the inflow and outflow velocities.

## 5. Effect of Age in CMR Studies

The age characteristics of the subjects and results of the CMR studies are summarized in Table 1 [14,30,33,46,47,48].

### 5.1. CMR Studies: Left Ventricle

Foll et al. [46] studied 24 healthy volunteers divided into 2 age groups: <30 and >50 years. The cut-off age was chosen based on the assumption that a large difference between the age-group cut-offs (20 years) would provide a clear separation between both groups. These authors observed variations in intracardiac flow dynamics with aging. Specifically, peak diastolic vortex velocities were significantly higher in the younger volunteers in the LV base compared to the older ones (69 ± 14 vs. 52 ± 16 cm/s; *p* = 0.005). Furthermore, age was inversely correlated to the velocity (r = −0.48, *p* = 0.02) of the basal LV vortices.

Crandon et al. [47] examined 53 healthy volunteers (mean age 45 ± 17 years) divided into 5 age groups. They observed that aging determines a decline in peak E-wave KEi_EDV_, an increase in peak A-wave KEi_EDV_, and a decrease in the KEi_EDV_ E/A ratio at higher ages. In particular, subjects aged over 50 years had a significantly lower KEi_EDV_ E/A ratio than all the other younger age groups. Correlations between peak E-wave KEi_EDV_, peak A-wave KEi_EDV_, and KEi_EDV_ E/A ratios with age were −0.51, 0.65, and −0.79 (*p* < 0.01), respectively. Crandon et al. [47] explained the progressive reduction in peak E-wave KEi_EDV_ with age through impaired LV myocardial relaxation. To compensate for impaired early diastolic LV filling, more filling would occur in late diastole, probably because of a stronger atrial contraction, thereby causing the peak A-wave KEi_EDV_ to increase. Gross (non-indexed) peak E-wave KE remained stable with age. All the other indexed KE parameters (LV systolic, diastolic, and global KEi_EDV_) did not show a significant association with age.

As expected, in the study of Crandon et al. [47], a strong correlation was observed between 2D mitral inflow metrics for Doppler echocardiography and 4D blood flow energetics using CMR. However, 4D flow KE metrics (KE of early and late mitral filling as well as KEi_EDV_ E/A ratio) demonstrated a stronger association with age, probably because these metrics are more closely associated with myocardial relaxation coupled with its resulting hemodynamic forces [47]. Indeed, 4D blood flow energy assessment not only includes mitral inflow but also the KE in the LV vortex, which, according to Crandon et al. [47], is plausibly associated with LV relaxation. Multivariate linear regression analysis demonstrated that both E/e’, a marker of myocardial relaxation, and the KEi_EDV_ E/A ratio were the most independently associated variables of aging.

Zhao et al. [30] studied 74 Asian subjects (mean age 42 ± 13 years) distributed in 5 age groups. These authors confirmed that the peak E-wave KEi_EDV_ and KEi_EDV_ E/A ratio both decrease, while the peak A-wave KEi_EDV_ increases with age. A sharp decline in peak E-wave KEi_EDV_ and an increase in peak A-wave KEi_EDV_ was observed in the ≥60 years age group. Zhao et al. [30] also confirmed a lack of significant differences in the LV global, diastolic, peak systolic, and systolic KEi_EDV_ with age. These latter data are consistent with the preservation of systolic function during healthy aging.

Garg et al. [14] compared two groups of healthy controls, a younger group (mean age 30 ± 10 years) and an older group (mean age 57 ± 7 years). The LV KEi_EDV_, which was averaged over the complete cardiac cycle, and the average systolic and diastolic KEi_EDV_ did not differ between the two groups. Conversely, in the older healthy subjects, the peak E-wave KEi_EDV_ and peak A-wave KEi_EDV_ were lower and higher, respectively. In this study, peak E-wave KEi_EDV_ and A-wave KEi_EDV_ were also computed for basal, mid, and apical LV levels by dividing the LV into equal thirds. The relative KE drop (in %) in the intraventricular E-wave and A-wave KE from base to mid-ventricle and from mid-ventricle to apex was calculated. This relative KE drop did not differ in the younger or older normal subjects.

Wong et al. [33] divided 35 healthy subjects (ranging in age from 1 to 64 years) into age quartiles. To allow a comparison between the hearts of different sizes, KE values were expressed as KE density, whereby KE was indexed to the LV volume at each time point during the cardiac cycle (KEi, in mcJ/mL). The authors found that healthy aging was associated with a fall in peak E-wave KEi. In particular, two distinct falls in the early diastolic KEi occurred: from childhood to adulthood, and especially in late adulthood, which was represented by the 4th quartile, and was aged 49 years and above. Wong et al. [33] hypothesized that changes in blood flow are the consequence of altered ventricular myocardial properties and that early diastolic KE represents a sensitive noninvasive marker of change. Actually, during healthy aging an increase in LV stiffness and a reduction in LV compliance occur [49], which become noticeable after 50 years of age [50,51]. Variations in stiffness or compliance may be due to an accumulation of collagen and increased extracellular matrix cross-linking [52,53,54].

### 5.2. CMR Studies: Right Ventricle

Barker et al. [48] studied blood flow within the RV of 53 normal subjects, aged between 20 and 80 years. Five age groups were considered. The authors found that with healthy aging RV peak E-wave KEi_EDV_ decreases, whereas RV A-wave KEi_EDV_ increases. Specifically, correlations with age were r = −0.30 (*p* = 0.04) for the RV E-wave KEi_EDV_, r = 0.42 (*p* < 0.01) for the A-wave KEi_EDV_, and r = −0.53 (*p* < 0.01) for the KEi_EDV_ E/A ratio. The RV global KE for the complete cardiac cycle was comparable in the different age groups and had no association with age. Moreover, the mean RV systolic and diastolic KEi_EDV_ did not show any significant association with aging. These results resemble those observed for the LV and suggest that RV diastolic filling adapts in a similar way to LV filling. Barker et al. [48] suggested that RV diastolic function worsened with aging due to the stiffness of the RV [55] and that the increase in RV peak A-wave KEi_EDV_ is compensatory to the decrease in RV E-wave KEi_EDV_. These results agree with the theory that an increase in right atrial contraction and pressure is required to maintain sufficient RV filling in older individuals [56].

Overall, the CMR studies performed on both ventricles show that the LV and RV KE evaluated during diastole varies with age [57]. This variation occurs at the time of the early and late ventricular filling, while an effect from age on the mean diastolic KE and LV non-indexed KE measures has not been clearly documented. Systolic and global KE have not been reported to vary with age for adults in either the LV or RV.

## 6. Effect of Age in EchoPIV Studies

There are no studies addressing the effect of age on intraventricular flow dynamics measures, specifically the evaluation of KE and KED using the Echo-PIV technique. Only small groups of normal subjects have been evaluated using this technique [41], probably because of the use of a contrast agent, which limits its application.

## 7. Effect of Age in Cardiac Ultrasound Studies

The age characteristics of the subjects and results of the cardiac ultrasound studies are summarized in Table 2 [28,29,44,58,59,60,61].

### 7.1. Ultrasound Studies: Left Ventricle

Compared to CMR studies, cardiac ultrasound techniques generally tend to evaluate KED/EL rather than KE because the former is a more reliable assessment, as stated before. Currently, reports exploring the relationship between age and EL have been based mainly on the evaluation of the LV using the VFM technique and in one case the BSI technique.

Chan et al. studied 100 healthy subjects (mean age 42.9 ± 14.9 years) divided into 3 age groups [59]. They observed that with normal aging, the peak E-wave EL (EL_E_) decreases and peak A-wave EL (EL_A_) increases, while the peak systolic EL (EL_S_) does not change. Both the mean diastolic EL (EL_D_) and mean EL_S_ did not change with age, which was interpreted as a sign of maintained energy efficiency in aging hearts. The mean EL_D_ was significantly higher than the mean EL_S_ across all age groups, which was attributed to the “U-turn” in the LV inflow at the LV mid-portion and apex, thereby causing turbulence in the LV that was related to a collision with the blood. In contrast, systolic blood flow converging in the normal LV outflow tract (LVOT) is unidirectionally forward in direction and is laminar. Here, the lack of flow collision limits intracardiac EL and determines EL_S_ preservation.

Adabifirouzjaei et al. [28] enrolled 101 volunteers, aged 20–80 years. They reported that EL during LV rapid diastolic filling (RF-EL) had a negative correlation with age (r = −0.592, *p* < 0.01), while EL, at the time of atrial contraction (AC-EL), had a positive correlation (r = 0.510, *p* < 0.01). The mean EL value during the full diastolic period, calculated as RF-EL + AC-EL/2, did not vary with age.

Akiyama et al. [58] studied 4 energy measures in 50 volunteers (mean age 29.5 ± 4.8 years). The first one of these measures was the mean KE over one cardiac cycle in the LVOT (KE_cycle_). The other three measures were the mean EL over one cardiac cycle (EL_cycle_), the mean systolic EL (EL_sys_), and the mean diastolic EL (EL_dia_). These authors also introduced a new index, the EPI (energetic performance index), defined as the ratio of KE_cycle_/EL_cycle_. In the univariate regression analysis, age was not significantly correlated to anyone in the explored energy measures, while it was significantly correlated to the mean EL_dia_ in the multivariate regression analysis (r = −0.582, *p* = 0.1479). It must be said, however, that the statistical significance in the multivariate analysis was set at *p* < 0.2, while in the univariate analysis, it was set at *p* < 0.05. The mean EL_dia_ was significantly correlated with the peak E-wave velocity (r = 0.542; *p* < 0.0001). EL was significantly higher in diastole than in systole, which was consistent with the results of Chan et al., as discussed above [59]. The effect of age on EPI was not evaluated.

Hayashi et al. [29] studied the mean systolic and diastolic EL values within the LV of 64 healthy children, aged 1–15 years. Subjects were divided in three age groups. The EL values were indexed to BSA, meaning that the absolute value of EL could be compared among the pediatric population with widely varied body sizes. These authors found that both the BSA-indexed mean systolic and diastolic EL had a strong negative correlation with age by univariate analysis (r = −0.80, *p* < 0.0001 for BSA-indexed mean systolic EL and r = −0.86, *p* < 0.0001 for BSA-indexed mean diastolic EL); however, this could be partly imputable to the indexing with BSA, which increased with age. In addition, the BSA-indexed mean diastolic EL directly correlated with the peak E-wave velocity (r = 0.42, *p* < 0.001). Using multivariate analysis, the age and heart rate were independent predictors of the BSA-indexed mean systolic EL, while age, heart rate, and E wave peak velocity were independent predictors of the BSA-indexed mean diastolic EL.

Becker et al. [60] enrolled 66 individuals (aged from 0 days to 22 years), including 14 subjects aged <2 months. The EL values were indexed to BSA. BSA-indexed peak and average systolic EL progressively decreased with age, with the highest values being in individuals aged <2 months. The BSA-indexed peak and average diastolic EL were initially low in subjects aged <2 months and increased when transitioning to the 2-month and 2-year age ranges; thereafter, they decreased after 2 years of age when transitioning to the 2–8-year age range and continued to decrease with patient age, with the overall lowest values occurring in adolescence.

Nyrnes et al. [44] applied the BSI technique in 51 normal control subjects (ranging in age from 2-day-old term neonates to 10-year-old children). The LV maximum diastolic KE and EL directly correlated to age (r = 0.73 and r = 0.72, respectively). These findings are in opposition to those by Hayashi et al. [29], who showed a negative correlation to age. Nyrnes et al. [44] did not use BSA-indexed values, while the age range was more limited, with the highest age being 10 compared to the highest age of 15 in the study by Hayashi et al. [29]. Moreover, the KE and EL measurements obtained by BSI in the study by Nyrnes et al. [44] showed high variance. All these factors may account, at least partly, for the discrepancy between the results of Nyrnes et al. [44] and Hayashi et al. [29].

### 7.2. Ultrasound Studies: Right Ventricle

Chen et al. [61] used VFM to study 90 healthy children aged 1–18 years, which were divided into 3 age groups. The average diastolic and systolic ELs were calculated for the RV in the RV-focused apical 4-chamber view and for the RV outflow tract and pulmonary trunk (OP) in the parasternal short-axis view. In the univariate analysis, a strong negative correlation was observed between the RV diastolic EL (r = −0.821, *p* < 0.001), RV systolic EL (r = −0.709, *p* < 0.001), OP diastolic EL (r = −0.597, *p* < 0.001), and OP systolic EL (r = −0.672, *p* < 0.001) and age. All these measures also correlated with the heart rate, although age was the main independent predictor in the multivariate linear regression analysis. Chen et al. [61] explained the strong correlation between RV and OP EL with age as the consequence of physiological alterations in cardiac contractility, which is enhanced when the heart rate increases due to the force–frequency effect. In other words, the increased cardiac contractility in subjects with younger ages or faster heart rates would generate more vortices in the RV and OP and lead to an increased diastolic EL [61]. The higher RV and OP systolic EL values at the youngest age agree with the results by Becker et al. [60] on the LV. Interestingly, Chen et al. [61] did not use BSA-indexed EL values.

In summary, ultrasound studies are concordant in describing a LV peak EL_E_ decrease and a peak EL_A_ increase in normally aged adults [28,59]. Some authors also reported a negative correlation in mean diastolic EL [58] and BSA-indexed mean systolic and diastolic EL with age [29]. However, other investigators did not observe a correlation between mean diastolic EL and age [28], and some researchers reported an unexpected direct correlation between LV maximum diastolic KE and EL with age in younger normal subjects [44]. In one study, a reduction in average systolic and diastolic RV EL with age was observed in healthy children [61].

## 8. Discussion

### 8.1. Effects of Aging on Ventricular KE and KED/EL

The popularity of the energetic measures of intraventricular blood flow, in terms of both KE and KED/EL, is increasing according to recent literature. Thus, there is a need to clarify the effect of physiological variables on these measures and specifically to elucidate the consequences of aging, a factor known to influence various indicators of cardiac function [21]. Overall, the CMR and echocardiographic studies published so far indicate that aging affects the energetics of left and right intraventricular blood flow (although not all energy measures during the cardiac cycle seem to be affected in the same way). This has physiological and pathophysiological implications. In particular, younger subjects and older adults deserve specific attention.

### 8.2. Ventricular Energetics in Younger Subjects

Becker et al. [60] observed a peculiar behavior in BSA-indexed peak and average LV diastolic EL in younger subjects. It was characterized by an increase in EL values when transitioning from newborns to the 2-month and 2-year age ranges. These authors evidenced that the increase in diastolic EL coincided with the development of a second counterclockwise early diastolic vortex in the LV, located at the level of the posterior mitral valve leaflet, which added to the clockwise vortex, initially observed at the anterior mitral valve leaflet level in newborns aged <2 months [60]. According to Becker et al. [60], the initial early diastolic anterior vortex helps in directing coordinated blood flow toward the cardiac apex during LV relaxation to maximize LV filling, minimize areas of blood-myocardial wall interaction and reduce shear stress and EL. The second early diastolic posterior vortex aids in maintaining coordinated blood flow within the growing LV. As the LV continues to increase in size, while the heart rate decreases with increasing age, stabilization of the second vortex allows for the progressive reduction in EL. These observations are coherent with results by Hayashi et al. [29] and Wong et al. [33].

In terms of peak and average LV systolic EL, a progressive decline in BSA-indexed EL values has been observed from newborns to adolescents [60]. A reduction in the average RV systolic EL has also been observed as the age increases in younger healthy subjects [61]. This phenomenon may be related to the reduction in heart rate, which has been recognized as a determinant of BSA-indexed systolic EL [29]. LV and RV systolic EL are expected to remain preserved during healthy aging in adult subjects [30,48].

### 8.3. Ventricular Energetics in Older Adults

Wong et al. [33] compared the physiological adaptations of LV KE occurring with healthy aging to the pathological alterations apparent in heart failure. These authors observed that the reduced early diastolic KE values in healthy older adults (20.6 ± 6.1 mcJ/mL) resemble the values seen in patients with heart failure and reduced ejection fraction (17.1 ± 6.3 mcJ/mL, *p* = 0.335). The observations of Wong et al. [33] raise the issue of whether the loss of LV early diastolic KE in healthy older adults should be considered a physiological or pathological phenomenon.

Interestingly, the same increase in cardiac stiffness associated with older age has been observed in patients >65 years with heart failure and preserved ejection fraction [62]. The age-related cardiac stiffness increase has been hypothesized to be a substrate for this type of heart failure [33], characterized by an excess in impaired relaxation [62]. According to Wong et al. [33], the diastolic KE could be used as a surrogate marker for LV compliance to better understand the pathophysiological mechanisms of heart failure with preserved ejection fraction.

In patients with heart failure and reduced ejection fraction, additional alterations have been described compared to healthy aging. These patients have a lower systolic KE (12.6 ± 5.0 vs. 17.8 ± 6.3 mcJ/mL in older adults, *p* = 0.033) and a borderline statistically significant increase in KE during diastasis (7.4 ± 4.0 vs. 3.6 ± 2.1 mcJ/mL in older adults, *p* = 0.051), which may be related to the altered filling mechanisms caused by ventricular dilatation [33]. As a consequence of these variations, a broadly flattened intracardiac KE profile occurs in patients with heart failure and reduced ejection fraction, which is not evident in healthy older adults.

### 8.4. Clinical Perspectives

Cardiac aging is characterized by a series of intricate events, including the development of LV fibrosis, diastolic dysfunction, and decreased maximal exercise capacity [23,24,25,26,27]. The aged myocardium is more vulnerable to stress and may be at least partly responsible for the high prevalence of cardiovascular diseases in the elderly. Mechanisms in the aging process are complex. They are genetically controlled and can be affected by environmental factors, which results in a variable rate of aging among individuals [23,24,25,26,27]. Recently, the so-called inflamm-aging has received particular attention [25]. Inflamm-aging describes the chronic, low-grade, systemic inflammation that develops with age in the absence of overt infections (sterile inflammation) [25]. Understanding the mechanisms underlying cardiac aging is the basis for emerging interventions, which target specific mechanisms to delay cardiac aging. These new therapeutic strategies could add to dietary interventions and supplementations [23,24,25,26,27].

**Figure 2:**
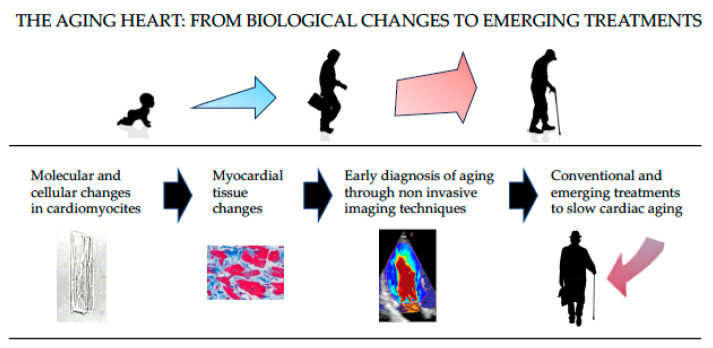
An illustration of the role in the early diagnosis of cardiac aging using noninvasive imaging techniques (i.e., intracardiac flow dynamics assessment) in the perspective of treatments finalized to delay aging.

Early recognition of cardiac aging is crucial (Figure 2). The analysis of intracardiac flow dynamics holds potential in this regard. The movement of blood through the LV is highly sensitive to changes in myocardial properties and is also additive to the evaluation of the diastolic transmitral inflow performed using conventional Doppler echocardiography. For example, in a study by Amaki et al. [63], energy dissipation differentiated normal from pseudonormal filling, even in the presence of a low E/e’ ratio. Furthermore, EL variations have been shown to recognize the effects of medical therapy in a dysfunctional heart [64], highlighting the value of intracardiac flow dynamics assessment as a potential clinical tool.

### 8.5. Limitations of Current Studies

The following considerations should be taken into account to correctly evaluate the results of the studies assessing the effect of aging on intraventricular KE and KED/EL.

Firstly, almost all the studies published so far rely on a limited number of subjects and have often small age subgroups. This has prevented the definition of age-related normal values for any energetic measure. Secondly, normal subjects in the older age groups are generally difficult to find and are under-represented in several studies compared to the younger groups of subjects. Older individuals are those in whom the age-related variations are expected to be more evident, meaning they should be better represented. Thirdly, most of the investigations were performed by the same groups of researchers, sometimes on the same individuals. Therefore, not only large but also multicenter studies are needed to avoid any investigational or statistical bias. Fourth, the evaluation of intraventricular energetic properties requires standardization of cardiac imaging techniques to avoid the influence of image resolution and acquisition/filtering settings. Furthermore, different cardiac imaging techniques should be compared using the same patients, to explore whether the results are coherent. Fifth, since other physiological variables might influence LV and RV KE and KED/EL measures (i.e., sex, ethnicity, heart rate, blood pressure, and exercise), multivariable analyses are needed to establish the relative weight of age with respect to the other potential independent predictors. Finally, there are no studies showing how LV and RV KE and KED/EL measures can guide treatment in patients with aged hearts, although early recognition of cardiac aging based on these measures can be a reasonable perspective, as described above.

### 8.6. Conclusions

Intraventricular KE and KED/EL are innovative measures that can potentially improve the characterization of cardiac function. These measures are affected by aging, although the current studies exploring the effect of age have limitations. Additional large, multicenter investigations are needed to test the effect of physiological variables on intraventricular KE and KED/EL measures.

## Figures and Tables

**Table 1:** Age characteristics of subjects and relevant findings of cardiac magnetic resonance studies. The studies are listed in the order of the year of publication. EDV: end-diastolic volume; KEi: indexed kinetic energy; LV: left ventricle; RV: right ventricle.

4D Flow Cardiac Magnetic Resonance Studies			
Left Ventricle			
Reference	N	Age characteristics	Most relevant findings
Föll et al. 2013 [46]	24	Two age groups: <30 years (*n* = 12, mean age: 23.3 ± 1.6 years, 6 women); >50 years (*n* = 12, mean age: 58.3 ± 4.2 years, 6 women).	Inverse correlation between age and velocity of basal LV vortices. Peak vortex velocities in the LV base are higher in younger subjects than in older ones.
Wong et al. 2016 [33]	35	Age range: 1 to 64 years. Mean age: 29 ± 13 years. Four age quartiles: < 16; 17–32; 33–48; 49–64 years.	Two falls in early peak E-wave diastolic KE_i_: from first to second quartile and from third to fourth quartile.
Crandon et al. 2018 [47]	53	Age range: 20 to 80 years. Mean age: 45 ± 17 years. Five age groups: 23 ± 2; 32 ± 3; 47 ± 4; 54 ± 2; 69 ± 6 years.	Decline in peak E-wave KEi_EDV_ and KEi_EDV_ E/A ratio and increase in peak A-wave KEi_EDV_ with age. KEi_EDV_ E/A ratio and E/e’ ratio are independently associated with aging.
Garg et al. 2019 [14]	40	Two age groups: group 1 (*n* = 24), mean age: 30 ± 10 years; group 2 (*n* = 16), mean age: 57 ± 7 years.	Peak E-wave KEi_EDV_ was lower and peak A-wave KEi_EDV_ was higher in the older subjects. No difference in LV global, average systolic, and average diastolic KEi_EDV_ with age.
Zhao et al. 2021 [30]	74	Age range: 20 to 80 years. Mean age: 42 ± 13 years. Five age groups: 20–29; 30–39; 40–49; 50–59; 60–80 years.	Over 60 years sharp decrease in peak E-wave KEi_EDV_ and KEi_EDV_ E/A ratio and increase in peak A-wave KEi_EDV_. Systolic function is preserved with aging.
Right Ventricle			
Reference	N	Age characteristics	Most relevant findings
Barker et al. 2020 [48]	53	Age range: 20 to 80 years (32 males, mean age: 41.5 ± 17 years, range 20–73; 21 females, mean age: 50.1 ± 16.8 years, range 27–80). Five age groups: 23 ± 2; 32 ± 3; 47 ± 4; 54 ± 2; 69 ± 6 years.	RV peak E-wave KEi_EDV_ and KEi_EDV_ E/A ratio decrease and RV A-wave KEi_EDV_ increase with age. Global KE is not associated with aging.

**Table 2:** Age characteristics of subjects and relevant findings of cardiac ultrasound studies. The studies are listed in the order of the year of publication. BSA: body surface area; BSI: Blood Speckle Imaging; EL: energy loss; dia: diastolic; KE: kinetic energy; OP: outflow tract and pulmonary trunk; RV: right ventricle; VFM: Vector Flow Mapping.

Cardiac Ultrasound Studies			
Left Ventricle			
Reference	N	Age characteristics	Most relevant findings
Hayashi et al. 2015 [29]	64	Age range: 1 to 15 years. Mean age: 6.8 ± 4.3 years. Three age groups: 1–5 (*n* = 34); 6–10 (*n* = 18); 11–15 years (*n* = 12).	VFM technique. BSA-indexed diastolic and systolic EL decrease with age. Age and heart rate independent predictors of mean systolic EL. Age, heart rate, and peak E-wave velocity are independent predictors of mean diastolic EL.
Akiyama et al. 2017 [58]	50	Age range: 20 to 44 years. Mean age: 29.5 ± 4.8 years. No age groups.	VFM technique. Mean EL_dia_ associated with age in multivariate, not univariate analyses.
Nyrnes et al. 2020 [44]	51	Age range: 2 days old to 10 years. Median age: 2.2 (0.1–5.6) years No age groups.	BSI technique. Direct correlation of KE and EL with age.
Chan et al. 2021 [59]	100	Age range: 18 to 67 years. Mean age: 42.9 ± 14.9 years. Three age groups: 18–29 (*n* = 26); 30–49 (*n* = 35); 50–67 (*n* = 39) years.	VFM technique. EL_E_ (peak E-wave EL) decreases, and EL_A_ (peak A-wave EL) increases with age. No change in peak and mean EL_S_ (systolic EL) and EL_D_ (mean diastolic EL) with age.
Adabifirouzjaei et al. 2021 [28]	101	Age range: 20 to 80 years. Mean age: 48.5 ± 16.6 years. Three age groups: 20–40 (*n* = 37); 41–60 (*n* = 32); 61–80 (*n* = 32) years.	VFM technique. Rapid filling-EL decrease and atrial contraction-EL increase with age. No change in mean EL (during the full diastolic period) with age.
Becker et al. 2023 [60]	66	Age range: 0 to 22 years Five age groups: 0–2 months (*n* = 14); 2 months-2 years (*n* = 7); 2–8 (*n* = 6); 8–13 (*n* = 21); 13–22 years (*n* = 18).	VFM technique. BSA-indexed peak and average diastolic EL increase between 2 months and 2 years and decrease in adolescence. BSA-indexed peak and average systolic EL progressive decrease with age.
Right Ventricle			
Reference	N	Age characteristics	Most relevant findings
Chen et al. 2019 [61]	90	Age range: 1 to 18 years. Mean age: 8.99 ± 5.35 years. Three age groups: 1–5 (*n* = 33); 6–10 (*n* = 25); 11–18 years (*n* = 32).	VFM technique. RV and OP average diastolic EL and RV and OP average systolic EL decrease with age.

## Data Availability

Not applicable.
